# Rapid Behavioral and Genomic Responses to Social Opportunity

**DOI:** 10.1371/journal.pbio.0030363

**Published:** 2005-10-18

**Authors:** Sabrina S Burmeister, Erich D Jarvis, Russell D Fernald

**Affiliations:** **1** Biological Sciences, Stanford University, Stanford, California, United States of America,; **2** Department of Neurobiology, Duke University Medical Center, Durham, North Carolina, United States of America; North Carolina State UniversityUnited States of America

## Abstract

From primates to bees, social status regulates reproduction. In the cichlid fish *Astatotilapia (Haplochromis) burtoni,* subordinate males have reduced fertility and must become dominant to reproduce. This increase in sexual capacity is orchestrated by neurons in the preoptic area, which enlarge in response to dominance and increase expression of gonadotropin-releasing hormone 1 (GnRH1), a peptide critical for reproduction. Using a novel behavioral paradigm, we show for the first time that subordinate males can become dominant within minutes of an opportunity to do so, displaying dramatic changes in body coloration and behavior. We also found that social opportunity induced expression of the immediate-early gene *egr-1* in the anterior preoptic area, peaking in regions with high densities of GnRH1 neurons, and not in brain regions that express the related peptides GnRH2 and GnRH3. This genomic response did not occur in stable subordinate or stable dominant males even though stable dominants, like ascending males, displayed dominance behaviors. Moreover, *egr-1* in the optic tectum and the cerebellum was similarly induced in all experimental groups, showing that *egr-1* induction in the anterior preoptic area of ascending males was specific to this brain region. Because e*gr-1* codes for a transcription factor important in neural plasticity, induction of *egr-1* in the anterior preoptic area by social opportunity could be an early trigger in the molecular cascade that culminates in enhanced fertility and other long-term physiological changes associated with dominance.

## Abstract 2

Cichlid fish can rise to dominance over subordinate males within minutes of the opportunity to do so; and this behavioural change is accompanied by changes in hypothalamic gene expression.

## Introduction

Among social animals, dominance can have long-term physiological consequences. For example, dominance status has been shown to control fertility [1–3], neurogenesis [4], growth rate [5], and stress physiology [6,7]. Although social regulation of physiology is a well-established phenomenon, little is known about the neural mechanisms linking the social environment to physiological changes associated with dominance. To understand these neural mechanisms, we study a cichlid fish, *Astatotilapia (Haplochromis) burtoni,* in which dominance is tightly coupled to reproductive physiology.

Among male *A. burtoni,* dominance status regulates reproduction at several levels, resulting in decreased fertility of subordinate males. Subordinate males have smaller, less-mature testes [3]; they lack a territory with a spawning site; they do not display the body coloration advertising dominance; and they infrequently perform dominance behaviors, such as territorial defense and courtship. Among vertebrates, such differences in reproductive maturation are controlled primarily by the hypophysiotropic gonadotropin-releasing hormone (GnRH) neurons in the basal forebrain [8]. In *A. burtoni* these neurons express *GnRH1* and are located in the anterior parvocellular preoptic nucleus (aPPn), which is the most anterior part of the preoptic area in teleosts; the preoptic area is a conserved brain region among vertebrates. In dominant males, the GnRH1 neurons in the aPPn have larger somas [9,10], higher *GnRH1* gene expression [11], and altered electrical properties [12] compared to subordinates. These differences in GnRH1 neurons are controlled by social status [10,13] and presumably underlie the differences in testes size. Other forms of GnRH, GnRH2 and GnRH3, are expressed in the midbrain tegmentum and the terminal nerve ganglion, respectively, but they do not appear to regulate reproductive physiology in this species [11,14,15], nor do they demonstrate socially induced neural plasticity in soma size or gene expression [11], although GnRH2 has been implicated in regulating reproduction in mammals [16].

Social cues regulate sexual maturation in many animals [17]. One remarkable feature of *A. burtoni* is that male reproductive capacity is socially regulated throughout life; in the adult, sexual capacity is reversible and, importantly, remains under the control of social cues. Since the number of dominant males at any given time is limited by territorial resources, rapidly growing subordinates frequently attempt to usurp territories from faltering dominant males, resulting in a dynamic social hierarchy [5]. When a male detects an opportunity to ascend in status, his body coloration and behavioral repertoire change first whereas changes in fertility lag behind [10,11]. Specifically, males becoming dominant have been shown to produce the behavioral and coloration aspects of dominance as early as 1 d following a change in the social environment, but they did not produce dominance behaviors at the rate of established dominant males until 7 d later [11]. Evident 7 d after a male ascends to dominance are increases in *GnRH1* expression, GnRH1 neuron soma size, and testes size [11]. The key question we address here is, how does social experience affect short-term changes in behavior and gene expression that can lead, ultimately, to changes in GnRH1.

To understand the early behavioral and genomic responses of subordinate males to an opportunity to ascend in status, we addressed two questions. First, what is the behavioral response to perception of social opportunity? Second, what is the genomic response to social opportunity in the aPPn and how does this relate to GnRH1 neurons? To answer these questions, we developed a novel behavioral paradigm in which resident subordinate males were provided an opportunity to ascend in status in their familiar home environments. Because we were interested in identifying early genomic responses that could regulate long-term physiological changes, we focused on *egr-1* (also called *zif268*, *NGFI-A*, and *zenk*), which codes for a transcription factor important for neuronal plasticity and links membrane depolarization to expression of late-response target genes [18–21]. We compared *egr-1* expression in the aPPn of ascending males to that of stable subordinate and dominant males in similar circumstances. To assess the specificity of the induction pattern we observed, we also examined *egr-1* expression in four control regions: the optic tectum (OT), a subdivision of the cerebellum, the terminal nerve ganglion, and the midbrain tegmentum. Our results reveal a potential molecular mechanism for translating social information into changes in fertility and contribute to the understanding of molecular and neurobiological mechanisms of social behavior.

## Results

Using activity-dependent genes to map functional responses of brain areas requires a paradigm in which behavioral changes can be measured on the same timescale as changes in gene expression and in which the behavioral context minimizes generalized disturbances that also induce gene expression that can confound results [22–24]. Because such studies had not been conducted before in fish, we developed a novel behavioral paradigm that met the requirements of functional genomic mapping (see Materials and Methods). This paradigm generated novel behavioral responses and allowed us to map the genomic consequences of social opportunity as described below.

### Perception of Social Opportunity

In our behavioral paradigm, we controlled the social experience of all subjects over a 14-d period that concluded in an observation tank with four females and one or two males (see Materials and Methods). The individuals in the observation tank could see, but not physically interact with, large communities of fish in adjacent tanks. We then gave some subordinate males an opportunity to ascend to dominance in this familiar home environment by removing a resident dominant male 1 hr before onset of lights (using infrared night-vision goggles). This allowed us to link perception of social opportunity to light onset and to do so in the absence of stressful physical disturbances; prior approaches involved multiple physical disturbances [9–11]. We compared these ascending males to (1) stable subordinate males that experienced identical circumstances except for removal of the suppressing dominant male and that, therefore, represent ascending males prior to social opportunity and (2) stable dominant males that expressed dominance in the same environment as ascending males but in the absence of new opportunity.

We found that subordinate males given an opportunity to ascend to dominance did so within minutes by changing their body coloration and behavior (Figure 1A–1D). Dominance coloration or behaviors were not displayed by stable subordinate males, which were in the continued presence of suppressing dominant males (Figure 1E); these males were behaviorally indistinguishable from ascending males prior to social opportunity (Figure 1C). Interestingly, we noted for the first time that stable dominant males did not display coloration aspects of dominance status before the lights came on, but re-expressed this trait along with dominance behaviors minutes after onset of lights (Figure 1F) with a similar latency to males undergoing social ascent (Figure 1D). This diurnal expression of body coloration in stable dominants suggests that the expression of dominance coloration is costly, and that it pays to express it only when receivers can see it. Furthermore, ascending males expressed dominance behaviors at similar rates as stable dominant males (Figure 1D versus 1F), in contrast to previous studies that observed dominance shifts occurring more gradually [11].

**Figure 1:**
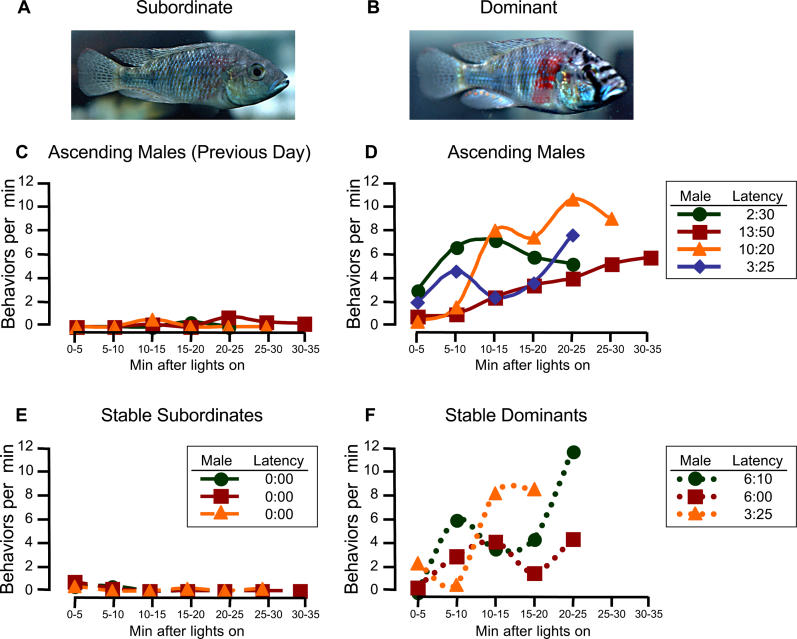
Time Course of Behavioral Response to Social Opportunity (A and B) Males displaying subordinate and dominant status. (C–F) Rate of dominance behaviors of individual ascending males the day before and during social opportunity, of stable subordinate males, and stable dominant males. Legends indicate latency (min:sec) to express dominance for each male on the day of sacrifice. Dominance behaviors are represented by composite scores consisting of the sum of chases, threat displays, courtship solicitations, and twice the number of spawning-site entries produced per minute.

Although ascending males expressed dominance with similar latency as stable dominant males, their behavioral responses were not identical. Compared to stable dominants, ascending males courted females slightly more, visited the spawning site slightly less, chased other fish at a similar rate, and threatened dominant males in the adjacent tank more frequently (Figure 2). In contrast, stable subordinates produced very few dominance-related behaviors (Figure 2), although they performed high rates of fleeing from the suppressing dominant males, a behavior not produced by ascending or stable dominant males (data not shown). Therefore, what differed between stable dominants and ascending males was the proportion of time spent producing a given dominance behavior; ascending males produced a higher proportion of threats toward neighboring dominant males.

**Figure 2:**
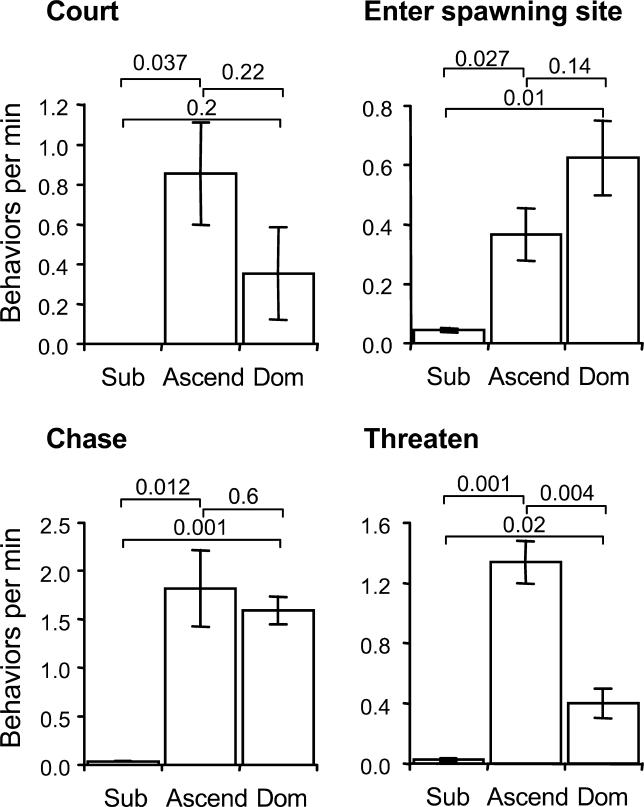
Behavioral Responses to Social Opportunity The rate of individual dominance behaviors per minute (mean ± SE) differed among stable subordinate (Sub), ascending (Ascend), and stable dominant (Dom) males. *p-*Values for individual comparisons are shown above bars.

To verify the reproductive status resulting from the prior 14-d social suppression on ascending and stable subordinate males, we calculated gonadosomatic index (GSI) as a measure of testes size relative to body size. Because GSI increases are only evident 1 wk after ascent [11], the 20 min of social dominance that ascending males experienced at the end of suppression is too short to cause increases in testes size. Socially suppressed males (mean GSI ± standard error [SE] = 0.46 ± 0.06) had smaller testes relative to their body size than stable dominant males (0.78 ± 0.22), although the difference was not statistically significant (*F_1,8_* = 3.86, *p* = 0.085). Because the GSI of suppressed and stable dominant males in our study was similar to that of long-term subordinate (0.43 ± 0.06) and dominant (0.74 ± 0.05) males, respectively [11], we concluded that the lack of a statistical difference in our study may be the result of small sample sizes.

### Rapid Genomic Response to Social Opportunity

We found that the rapid behavioral responses to social opportunity were matched by a rapid genomic response in the aPPn: Males ascending to dominance had a greater than 2-fold induction of *egr-1* compared to stable dominant and stable subordinate males (Figure 3). The stable dominants and stable subordinates did not differ from one another. The genomic response in the aPPn of ascending males was anatomically heterogeneous: induction was greatest in the central region (sections 4 and 5, approximately 168–224 μm caudal of the anterior commissure), less strong in the flanking regions (sections 1–3 and 6–7), and undetectable in the caudal-most region (sections 8–10; Figure 3B). To determine whether the lack of *egr-1* response in the caudal-most sections could be the result of an inherent inability of this region to induce *egr-1,* we compared the *egr-1* levels of males in the experiment to a positive control male that had been injected with a glutamate receptor agonist, kainic acid, which induces high levels of *egr-1* in the aPPn and elsewhere [25]. In the caudal-most region of the aPPn (sections 8–10), the kainic acid–injected male had 66% higher *egr-1* levels than ascending males (Figure 3C). A negative control male, which was sacrificed minutes before onset of lights, had the lowest level of *egr-1* expression (Figure 3C). Thus, the lower levels of *egr-1* in the caudal aPPn of ascending males cannot be explained by an inherent inability of this region to express higher levels of *egr-1,* and suggests that *egr-1* induction in the anterior part of the aPPn of ascending males represents a specific response rather than a general increase in *egr-1.*

**Figure 3:**
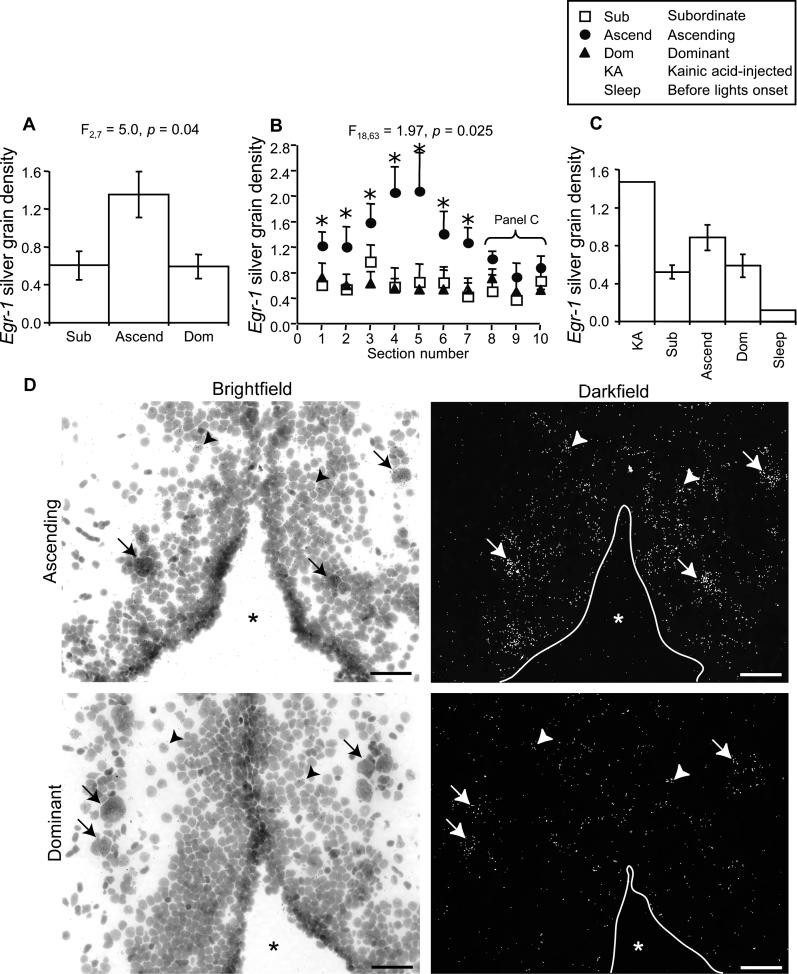
egr-1 Induction in the aPPn (A) Mean ± SE silver grain density of the entire aPPn differed among subordinate (Sub), ascending (Ascend), and dominant (Dom) males. (B) Induction of *egr-1* (mean ± SE silver grain density) in the aPPn of ascending males varied with section number (rostral to caudal) as compared to subordinate and dominant males. Asterisks represent contrasts with *p* < 0.05. (C) Mean ± SE silver grain density in the three caudal-most sections of the aPPn of subordinate, ascending, and dominant males compared to a male injected with kainic acid (KA) and a male sacrificed just before onset of lights (Sleep). (D) Photomicrographs of *egr-1* expression in the aPPn of an ascending male (top) and a stable dominant male (bottom). We chose representative males and sections to be close to group means for the central aPPn. Arrows indicate examples of *egr-1*-expresssing GnRH1 neurons identified by their distinctly large cellular morphology, arrowheads indicate examples of non-GnRH1 neurons, and the asterisks indicate the preoptic recess of the third ventricle. Scale bars represent 40 μm.

To further characterize the specificity of the response in the aPPn, we measured *egr-1* expression in two control regions: a sensory region, the OT, which integrates visual and somatosensory information, and a motor region, the torus longitudinalis (TL), a subdivision of the teleost cerebellum, which integrates visual information with motor output. Although *egr-1* was expressed in these brain regions, the expression levels did not vary with social status or social opportunity (Figure 4), nor was there a significant interaction between group and brain section. Social opportunity, therefore, regulated induction of *egr-1* in the aPPn, but not in these sensory and motor regions.

**Figure 4:**
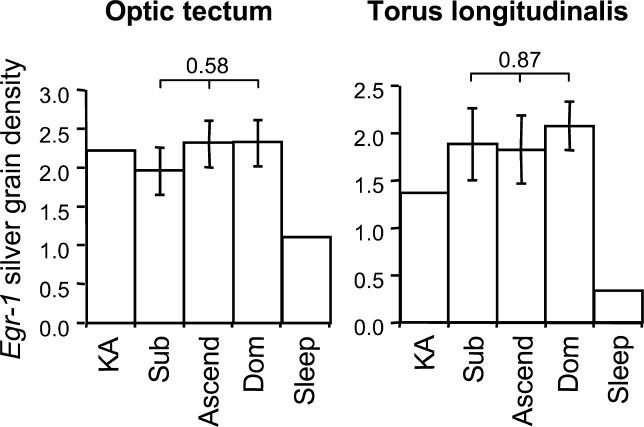
egr-1 Induction in the OT and TL Mean ± SE silver grain density in the OT or the TL did not vary among subordinate (Sub), ascending (Ascend), and dominant (Dom) males. The *p*-values for the effect of social opportunity are shown above bars. Shown for comparison is a male injected with kainic acid (KA) and a male sacrificed just before onset of lights (Sleep).

To determine whether the neuroanatomical heterogeneity of *egr-1* induction in the aPPn was related to the distribution of GnRH1 neurons, we compared the aPPn *egr-1* response of ascending males with the number of neurons expressing *GnRH1* on adjacent brain sections. Our *GnRH1* probe was specific to *GnRH1,* which codes for the hypophysiotropic form of GnRH in this species [26]. We found that aPPn *egr-1* induction was greatest in sections corresponding to those that had largest numbers of GnRH1 neurons and that this relationship accounted for 86% of the observed variation in *egr-1* induction (Figure 5A and 5B). The increase in *egr-1* expression in regions with high numbers of GnRH1 neurons is not due to generalized differences in cell density; our measure of *egr-1* induction, silver grain density, took into account such differences. We determined whether the *egr-1* was co-expressed with *GnRH1* by performing a double-label in situ hybridization. We found that *egr-1* was expressed in both *GnRH1*-expressing and non-*GnRH1*–expressing neurons (Figure 5C). We quantified the degree of double labeling among ascending males and found that the majority of GnRH1 neurons (78.5%) expressed *egr-1* with a population median of 15 co-localized *egr-1* silver grains (Figure 5D). Taken together, these data demonstrate that the region of the aPPn with the highest density of GnRH1 neurons was most responsive to social opportunity.

**Figure 5:**
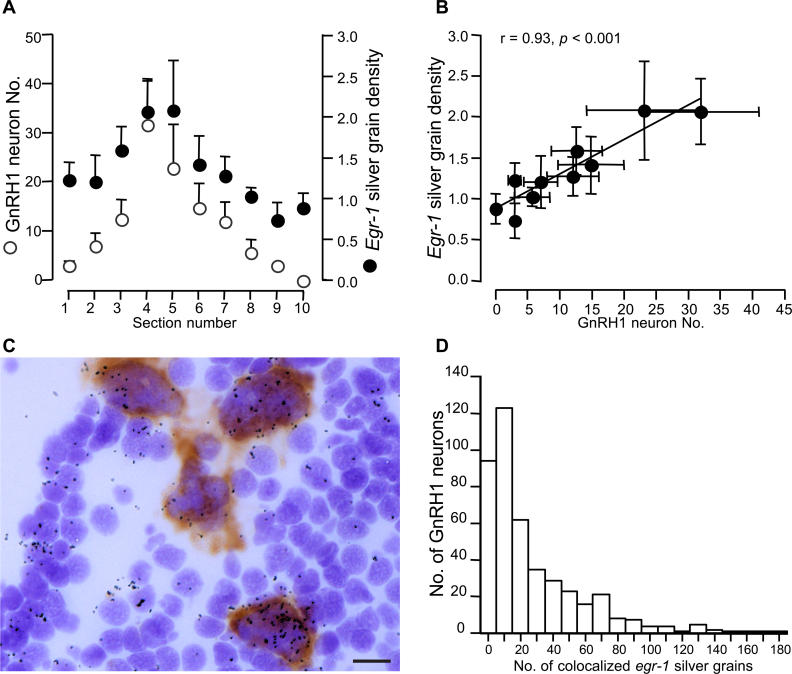
Relationship between *egr-1* and GnRH1 in the aPPn (A) Mean + SE GnRH1 neuron number (indicated by an open circle) and *egr-1* silver grain density (indicated by a closed circle) by section number (rostral to caudal). (B) Covariation of mean ± SE GnRH1 neuron number and *egr-1* silver grain density within sections. (C) Photomicrograph of *egr-1* (silver grains) and *GnRH1* (brown precipitate) expressing neurons in the aPPn. Scale bar represents 10 μm. (D) Histogram showing the number of GnRH1 neurons with the corresponding range of co-localized *egr-1* silver grains.

As a comparison, we also examined expression of *egr-1* in the non-hypophysiotropic GnRH neuron populations, which do not demonstrate socially induced plasticity. We found that neither the *GnRH2*-expressing midbrain tegmentum neurons nor the *GnRH3*-expressing terminal nerve ganglion neurons expressed *egr-1*, even in the kainic acid–injected male (data not shown), suggesting that these cell populations are not capable of expressing *egr-1*. We determined that the *A. burtoni GnRH1* gene is a candidate target of the egr-1 transcription factor by localizing an egr-1 binding site ( GCGGCGGCG [27]) 1,311 nucleotides upstream of the transcription start site [28]. Neither the *GnRH2* nor the *GnRH3* genes contain a putative egr-1 binding site.

## Discussion

Using a novel behavioral paradigm, we found that the behavioral transformation from subordinate to dominant can occur within minutes, and that this transformation is associated with a rapid genomic response in the aPPn, the most anterior nucleus of the preoptic area. Below we describe the behavioral and physiological implications of our findings.

### Perception of Social Opportunity

We found that ascending males rapidly adopted dominance coloration and produced the full array of dominance behaviors at rates that were similar to or higher than stable dominant males. The main difference we found was a greater number of threat displays produced by ascending males. Ascending males may have emphasized threat displays more than stable dominants in order to assert their new status with dominant males in neighboring tanks. Stable dominant males, in contrast, may have been less likely to be challenged by their familiar neighbors (i.e., the “dear enemy” phenomenon [29,30]) and could, therefore, invest more time engaging in reproductive behaviors, such as maintenance of the spawning site.

Perception of social opportunity requires a sophisticated integration of social cues because the information inherent in social opportunity is conveyed by the absence of a dominant male. The dominant male's absence is salient because it provides information about the focal male's future relationship to others within his complex social group. The response of ascending males suggests that they perceive the dominant male's absence as an opportunity to ascend in status.

The shift to dominance by ascending males within minutes of social opportunity demonstrates a social perspicacity previously unknown to exist in this species. The most detailed prior time course of behavioral responses to social opportunity measured changes over the course of days [11]. White et al. [11] reported that when subordinate males are moved to a new community where they are the largest, they increase their dominance within 1 d and continue to increase their levels of dominance until they are indistinguishable from stable dominant males 7 d later at the earliest. In contrast, we found that ascending males expressed dominance behaviors at rates similar to or greater than stable dominants within 14 min of social opportunity. Although no previous studies have reported the behavioral response to social opportunity on a timescale of minutes, in our personal experience, subordinate males do not ascend to dominance within 30 min of social opportunity when they are moved into a new community, the method used in prior studies [9–11]. Rather, when a male is caught and moved into a new environment, his initial response is to produce escape behaviors. The pronounced reduction in response time in our study is likely due to our method of presenting social opportunity that minimized disturbance to the focal subordinate male.

Social history may be another potentially important factor contributing to rapid responses to social opportunity. In our paradigm, subordinate males had been dominant 2 wk before, which differs from previous studies [9–11] that used males who had been subordinate for longer periods of time prior to social opportunity. Although there is no known mechanism for recent experience priming responses to social opportunity, we speculate that such a priming effect may enable males to respond quickly to rapidly changing social situations in nature. In a constant environment in the laboratory, males adopt stable social relationships that go unchanged for 7 to 9.5 wk on average [5]. However, fluctuating environments designed to mimic nature greatly shorten status tenure so that males remain subordinate for 4 wk and dominant for 3 wk on average [5]. Our study more closely resembled the fluctuating environment in terms of status tenure and, as such, may more closely resemble natural circumstances. If so, this would suggest that in naturally dynamic social situations, males are able to respond to socially opportunity without delay.

### *egr-1:* Linking Social Opportunity to Reproductive Function

We found that males ascending to dominance induced high levels of *egr-1* in their aPPn compared to stable subordinates and dominants. Stable dominant males had low *egr-1* expression in the aPPn at the same level as stable subordinates, despite the fact that stable dominants, like ascending males, were expressing dominance. Because *egr-1* induction may decline with repeated exposure to a stimulus [31], it is worth emphasizing that dominant males had not been expressing dominance for the previous 12 hr of darkness; they re-expressed dominance after lights-on, but did not induce *egr-1* to high levels in the aPPn. These findings suggest, therefore, that *egr-1* induction in the aPPn of ascending males is not a product of dominance per se, but a product of social opportunity.

Social opportunity is a complex experience that includes the perception of opportunity and the behavioral response to it. We wondered if the *egr-1* induction by social opportunity could be explained by the simple sensory or motor aspects of the experience. Since the sensory recognition of social opportunity requires a complex integration of social cues, which is mediated by the absence of a conspecific, induction of *egr-1* in the aPPn is unlikely to be explained by simple sensory aspects of the experience. This is consistent with the idea that the preoptic area is not a primary sensory station, but integrates internal and external information to regulate motivation and physiology [32]. The behavioral motor response to social opportunity is another possible candidate for mediating socially-induced *egr-1* expression in the aPPn because ascending males produced more threat displays than did stable dominants. However, we note that the ascending male with the highest *egr-1* produced the fewest threat displays of this group (data not shown). Further, stable dominant males produced intermediate numbers of threat displays, whereas the subordinate males produced no more than one, yet their *egr-1* expression levels in the aPPn were the same. From these data, it is difficult to conclude that there is a simple relationship between the motor production of threat displays and aPPn *egr-1* expression. The more interesting possibility is that threat displays regulate *egr-1* expression only in the context of social opportunity. Indeed, there is precedence for social context causing large differences in motor-driven *egr-1* expression [24,33], suggesting that the stimulus context itself, or subtle behavioral motor differences between the two contexts, are able to cause large differences in *egr-1* expression. Similarly, past social context is known to influence subsequent *egr-1* expression [34,35]. In summary, we propose that induction of *egr-1* in the aPPn by social opportunity is the result of a complex integration of social signals that transcends simple sensory or motor aspects of the experience.

*egr-1* expression in the aPPn is a potential link between social opportunity and enhanced reproductive function. The e*gr-1* induction in ascending males peaked in regions neuroanatomically associated with GnRH1 neurons. *egr-1* was not expressed in the non-hypophysiotropic GnRH2 and GnRH3 neurons that lack socially induced plasticity [11]. Although neuronal egr-1 targets regulated by physiological stimuli in vivo have yet to be identified, candidate targets in vitro include synapsins [36,37] and neurofilaments, among others [20]. Such target genes may contribute to the dramatic structural enlargement that occurs in the GnRH1 neuron somas over the course of 7 d [11]. In addition, we determined that the *GnRH1* gene, but not *GnRH2* or *GnRH3*, contains a binding site for the egr-1 transcription factor. Thus, the coupling of aPPn *egr-1* expression to social cues may be a mechanism for initiating structural changes in aPPn neurons, as well as changes in *GnRH1* expression itself. Our finding that social information is transduced into molecular changes by *egr-1* reveals a potential molecular mechanism for translating social information into changes in fertility and contribute to a growing understanding of social regulation of reproductive physiology [38–40].

## Materials and Methods

### Social manipulation

All subjects were adult males (6.75–8.25 cm standard length) raised from our laboratory stock that was originally derived from a wild population in Lake Tanganyika, Africa. All procedures were approved by the Stanford University Administrative Panel on Laboratory Animal Care committee. To perform behavioral molecular mapping with activity-dependent gene induction, we required a behavioral paradigm that would minimize disturbance of the social interactions and physical environment. Previous studies manipulated social environment by moving subordinate males to a new physical and social community (e.g., [11]) which may cause activation of gene regulation in many brain areas, and also inhibit rapid expression of dominance due to stress and/or the challenges of learning a new environment. Thus, we developed a paradigm where we tested a male's response to social opportunity in a familiar social and physical environment. To create a social opportunity, we removed a resident dominant male, rather than moving the subordinate male of interest. We paid close attention to the relative sizes of individuals, and we minimized the time spent in the socially reduced environment needed to produce a social opportunity. We minimized disturbance of the subjects by visually isolating the experimental observation tank with a black curtain.

To control for recent social experience, we began with males who were identified by focal observations as dominant (total *n* = 10). We then placed these males in different social environments to manipulate their status. In group 1 (“ascending males,” *n* = 4), males were socially suppressed for 14 d in a two-stage process and then given an opportunity to ascend in status on day 15. We chose 14 d of social suppression because this is sufficient to suppress the reproductive axis as measured by testes size. In stage 1 of social suppression (days 1–12), males were transferred from their original home tank where they were dominant to a social suppression tank that contained multiple larger dominant males, other subordinate males, and females. In stage 2 (days 13–14), these males were transferred to an experimental observation tank isolated by a curtain, which contained one larger dominant male who had previously established his territory in the tank (the suppressor) and four females. During both stages of social suppression, we used focal observations to confirm that subjects maintained their subordinate status. We used this two-stage approach to minimize the amount of time spent in the reduced social environment of the observation tank because extended periods in dominant–subordinate dyads can lead to injury of subordinate males. The reduced social circumstance of a dominant–subordinate dyad was necessary to control which individual ascends in status following removal of the dominant male. The observation tank was adjacent to two community tanks separated by transparent barriers; water circulation was shared among compartments. The adjacent community tanks contained multiple dominant males, subordinate males, and females that were all smaller in size than the males in the observation tank. Males visually interacted with other males across the transparent barrier. We reasoned that the subordinate subjects would be more inclined to ascend in status upon the removal of the suppressor if he perceived himself to be the largest male among those males he could see.

On the morning of day 15, group 1 subjects were given an opportunity to ascend to dominance by the removal of the suppressor 1 hr before onset of the lights. We removed the suppressor in the dark with the aid of infrared night-vision goggles. This minimized the disturbance to the subjects by preventing them from seeing the experimenter or the net in the dark, and preventing them from detecting the absence of the suppressor until the lights were turned on. Because *egr-1* gene expression peaks at approximately 30 min following stimulation [25], we sacrificed group 1 males 20 min after they produced one of two behaviors that are typical of dominance: a threat display directed toward a dominant male in an adjacent community tank or three rapid sequential chases of the females in the observation tank (dominant males will chase other fish, including females, away from their territories unless engaged in courtship). We recorded behavior with a digital video camera the morning before (14th day) and morning of (15th day) sacrifice, beginning with the onset of lights, for later quantification.

In group 2 (“stable subordinates,” *n* = 3), we created stable subordinate males by socially suppressing males for 14 d in a two-stage process identical to that for group 1 males. On the morning of the 15th day, we simulated removal of the suppressor by dipping a net into the tank 1 hr before onset of the lights, and, beginning with the onset of lights, we recorded behavior with a digital video camera for later quantification. Because we did not remove the suppressor, group 2 males remained subordinate (see Results). Each male in group 2 was paired with a subject in group 1 and sacrificed at the same time relative to onset of lights.

In group 3 (“stable dominants,” *n* = 3), we created stable dominant males by leaving the dominant males in their original home tanks during the first 12 d of the experiment. On day 13, these dominant males were put into the observation tank containing four females and no other males. On the morning of the 15th day, we simulated removal of a fish by dipping a net into the tank 1 hr before onset of the lights, and, beginning with the onset of lights, we recorded behavior with a digital video camera for later quantification. To determine time of sacrifice for group 3 males, we used identical criteria to that of group 1 males. That is, we sacrificed them 20 min after they first produced an aggressive dominance behavior.

To verify the success of reproductive suppression of males in groups 1 and 2 compared to males in group 3, we measured body mass and testes mass at the time of sacrifice to calculate GSI (testes mass divided by body mass multiplied by 100) as a measure of relative testes size.

In addition to males who underwent social manipulation, we sacrificed two males for use as a positive and negative control in the *egr-1* in situ hybridization. For the negative control, we sacrificed a dominant male minutes before onset of lights. For the positive control, we injected a male with kainic acid (10 mg per kg body mass) and sacrificed him 30 min later. A prior study [25] demonstrated that kainic acid causes an up-regulation of *egr-1* in the aPPn, and males sacrificed before onset of lights have dramatically reduced *egr-1* expression throughout the brain.

We quantified four behaviors (see [41] for details): two agonistic behaviors (chases and threat displays) and two reproductive behaviors (courtship solicitations and spawning-site entries). We defined chases as the subject forcibly swimming toward another fish (directed toward males and females). We defined threat displays (directed toward males) as lateral displays or border threats. We defined courtship solicitations (directed toward females) as presentation of the anal-fin egg spots and leading, whereby males invite the female to the spawning site. We defined spawning-site entries as any time the male entered the spawning site which, in the laboratory, is an over-turned pottery shard; the pot is where spawning would take place and it defines the center of a male's territory. We recorded the latency to express dominance as the latency to perform a threat display or three rapid chases after onset of lights. We calculated the rate of each behavior (behavior per minute) within each 5-min bin from lights-on to sacrifice. For group 1, we also quantified these behaviors the day before social transition to verify that they were subordinate before transition. To graphically display the development of dominance in Figure 1, we calculated a composite score, “dominance behavior,” as the sum of chases, threat displays, courtship solicitations, and twice the number of spawning-site entries produced per minute within each 5-min bin. We multiplied the number of spawning-site entries by two in our composite score because this is a low-frequency behavior compared to the others, but is highly significant.

### Analysis of *egr-1* expression in situ

Males were sacrificed by rapid decapitation, and we removed, froze, and stored their brains at −80 °C. We sectioned brains in three alternate series at 14 μm in the transverse plane. To detect *egr-1* mRNA, we followed published radioactive in situ hybridization (ISH) procedures [25], dipped the slides in emulsion, processed them for autoradiography, and counterstained them with cresyl violet. We quantified expression of *egr-1* by calculating the density of silver grains above cell bodies (number of silver grains per total cell area in pixels). Our method measures silver grain density for all cells in the field of view, which we estimated to be 350–1,250 cells per image (mean 830) for the aPPn, 485–2,535 cells per image (mean 1,322) for the OT, and 1,110–2,283 cells per image (mean 1,742) for the TL. For the aPPn, we calculated mean silver grain density of ten sequential sections (each separated by 42 μm) for each subject beginning with its origin just caudal to the anterior commissure. For the OT, we calculated mean silver grain density of four sequential sections (each separated by 42 μm), taking images of the left and right hemispheres separately and beginning with the most anterior sections in which the periventricular layer appeared. In the OT, we focused on the periventricular layer because it contains the cell bodies of neurons receiving incoming visual and somatosensory information. For the TL, we calculated mean silver grain density of four sections (each separated by 126 μm), taking images of the left and right hemispheres separately and beginning with the origin of the TL.

We used an image analysis strategy modified from published reports [42–44] as follows. We captured three images per brain section using a 40× objective and digital camera (Spot Camera, Diagnostic Instruments, Sterling Heights, Michigan, United States): one with a blue filter to de-emphasize cell bodies relative to the black silver grains, a second with a green filter to emphasize cell bodies, and a third image of the glass slide next to the brain section (with blue filter) to quantify the background density of silver grains. We converted all images to gray scale before analysis with ImageJ (National Institutes of Health, Bethesda, Maryland, United States) as follows. To measure the area covered by cells in the green image, we applied a binary threshold, filtered out small objects, measured the mean gray value of the image, and then converted that value to total cell area in pixels. We then added a 3-pixel halo to cells in the cell image in order to include silver grains near cell bodies. To quantify silver grains, we applied a binary threshold to the blue image and subtracted the binary cell image (including halo) to remove silver grains that did not occur above or near cell bodies. We then counted particles. For the blue background image, we similarly counted particles and calculated the area covered by silver grains without subtracting the binary cell image. We then calculated silver grain density per total cell area and subtracted from this the silver grain density of the background image. In the figures, *egr-1* expression levels are represented as silver grains per cell area in pixels multiplied by 1,000.

### Double-label ISH for *egr-1* and *GnRH1*

To localize *GnRH1*-expressing neurons and to determine whether *egr-1* and *GnRH1* are co-expressed, we used a second series of sections for a double-label ISH combining isotopic *(egr-1)* with digoxygenin *(GnRH1)* detection. Our *GnRH1* probe (188 base pairs, corresponding to nucleotides 18–206) was specific to *GnRH1,* which codes for the hypophysiotropic GnRH in this species. As part of a separate study, we also included a digoxygenin-labeled probe for arginine vasotocin, which is expressed in cells neuroanatomically and morphologically distinct from *GnRH1*-expressing cells [45]. The procedure for double detection was similar to the ISH for *egr-1* alone, except for the following changes: Sections were fixed for 10 min, we included the *GnRH1* probe (1 ng/μl) during hybridization which was carried out at 60 °C, RNAse treatment was reduced to 10 min at room temperature, high stringency washes were at 60 °C for 20 min each (once in 50% formamide and 2× SSC, twice in 0.1× SSC). After the 5-min room temperature 0.1× SSC wash, we proceeded with detection of the digoxygenin-labeled *GnRH1* probe as follows. We washed for 5 min in PBS, quenched endogenous peroxidases in 3% H_2_O_2_ (10 min), washed 5 min each in PBS and PBS with 0.3% tween (PBSTw), blocked for 30 min in blocking solution (Perkin-Elmer, Boston, Massachusetts, United States), incubated with anti-digoxygenin conjugated with HRP (1:250) for 2 hr, washed twice in PBSTw (5 min), incubated in TSA (1:50; Perkin-Elmer) for 5 min, washed twice in PBSTw (5 min), incubated for 30 min in avidin-HRP (1:100), washed in PBSTw and PBS (5 min each), incubated in diaminobenzidine (0.05%) with H_2_O_2_ for 5 min, washed twice in PBS (5 min each), and dehydrated in increasing concentrations of alcohol. After exposure to film, slides were dipped in emulsion (1:1 dilution in distilled water; Kodak, New Haven, Connecticut, United States) and stored for 4.5 d before developing and counterstaining with cresyl violet. Sections that were hybridized with a sense probe did not show binding detectable above background.

To quantify the distribution of *GnRH1-*expressing neurons in the aPPn, we used a 100× objective to identify and count the total number of GnRH1 neurons in each section. In addition, to assess the degree of double-labeling of *GnRH1* and *egr-1,* we counted the number of silver grains above *GnRH1*-expressing neurons among ascending males. We used a threshold of five silver grains, at or above which we counted a GnRH1 neuron as expressing *egr-1*. However, due to a problem with the emulsion separating from some slides, we were unable to assess the degree of double-labeling in stable subordinate or dominant males.

### Statistics

To test for differences in the rate of specific behaviors, we used an analysis of variance (ANOVA) for each behavior separately (chase, threaten, court, and enter spawning site) followed by multiple pairwise *t-*tests. To determine the effect of social suppression on testes size, we compared stable dominant males to males that were suppressed (stable subordinates and ascending males) using ANOVA. We used three two-way repeated measures ANOVAs to test for an effect of social opportunity on *egr-1* levels in the aPPn, OT, and TL, with group as a between-subjects factor and section number as the within-subjects factor. The main effect for group compares mean *egr-1* levels across groups whereas the interaction (group × section) determines whether this induction pattern varied neuroanatomically. To determine in which sections ascending males showed *egr-1* induction in the aPPn, we compared ascending males to stable males (dominants and subordinates considered together) using a repeated-measures ANOVA with least significant difference post hoc contrasts. Among ascending males, we used Pearson's correlation to assess the relationship between aPPn *egr-1* levels and GnRH1 neuron number in corresponding sections. All ANOVAS used Type III sums of squares, and for all statistical tests we evaluated the likelihood of an effect using the *p*-value.

## Supporting Information

### Accession Numbers

The GenBank (http://www.ncbi.nlm.nih.gov/Genbank) accession numbers for the genes and genetic material discussed in this paper are *egr-1* mRNA (AY493348), *GnRH1* mRNA (U31865), *GnRH1* gene (AF076961), *GnRH2* gene (AF076962), and *GnRH3* gene (AF076963).

## Abbreviations

ANOVA: analysis of variance
aPPn: anterior parvocellular preoptic nucleus
GnRH: gonadotropin-releasing hormone
GSI: gonadosomatic index
ISH: in situ hybridization
OT: optic tectum
SE: standard error
TL: torus longitudinalis
